# Involvement of TRPM2 and TRPV1 channels on hyperalgesia, apoptosis and oxidative stress in rat fibromyalgia model: Protective role of selenium

**DOI:** 10.1038/s41598-017-17715-1

**Published:** 2017-12-13

**Authors:** Esra Yüksel, Mustafa Nazıroğlu, Mehmet Şahin, Bilal Çiğ

**Affiliations:** 1 Division of Rheumatology, Department of Internal Medicine, Faculty of Medicine, Suleyman Demirel University, Isparta, Turkey; 2 Neuroscience Research Center, Suleyman Demirel University, Isparta, Turkey; 3 Department of Biophysics, Faculty of Medicine, Suleyman Demirel University, Isparta, Turkey; 4 Department of Neuroscience, Institute of Health Sciences, Suleyman Demirel University, Isparta, Turkey

## Abstract

Fibromyalgia (FM) results in pain characterized by low selenium (Se) levels, excessive Ca^2+^ influx, reactive oxygen species (ROS) production, and acidic pH. TRPM2 and TRPV1 are activated by ROS and acid; nevertheless, their roles have not been elucidated in FM. Therefore, we investigated the contribution of TRPM2 and TRPV1 to pain, oxidative stress, and apoptosis in a rat model of FM and the therapeutic potential of Se. Thirty-six rats were divided into four groups: control, Se, FM, and FM + Se. The Se treatment reduced the FM-induced increase in TRPM2 and TRPV1 currents, pain intensity, intracellular free Ca^2+^, ROS, and mitochondrial membrane depolarization in the sciatic (SciN) and dorsal root ganglion (DRGN) neurons. Furthermore, Se treatment attenuated the FM-induced decrease in cell viability in the DRGN and SciN, glutathione peroxidase, and reduced glutathione and α-tocopherol values in the DRGN, SciN, brain, muscle, and plasma; however, lipid peroxidation levels were decreased. Se also attenuated PARP1, caspase 3, and 9 expressions in the SciN, DRGN, and muscle. In conclusion, Se treatment decreased the FM-induced increase in hyperalgesia, ROS, apoptosis, and Ca^2+^ entry through TRPM2 and TRPV1 in the SciN and DRGN. Our findings may be relevant to the elucidation and treatment of FM.

## Introduction

Fibromyalgia (FM) is a common chronic pain syndrome affecting up to 4 million adults in the United States, about 2% of the adult population^1^. Various factors such as oxidative stress and calcium ion (Ca^2+^) influx overload play major roles in the etiology of FM. Several pharmaceutical drugs such as antidepressants and voltage-gated calcium channel blockers are recommended for the treatment of FM; however, they fail to produce a satisfactory response in patients with FM because of the unclear etiology of the disease^2,3^. Therefore, it is highly important to elucidate the etiology of FM and develop more effective therapeutic strategies. The injection of acidic solutions into the gastrocnemius muscle of rats can produce a bilateral long-lasting hyperalgesia similar to FM in humans^4^.

The activation of acid-sensitive cation channels may contribute to pain induction in FM^5,6^. Indeed, acidosis from lactate accumulation and oxidative stress from the ischemia of muscles and tender points are two common triggers of pain in patients with FM^6,7^. Therefore, FM is strongly associated with acid-sensing and oxidative stress-sensing cation channels. Transient receptor potential (TRP) melastatin 2 (TRPM2) and vanilloid 1 (TRPV1) are two members of the Ca^2+^-permeable TRP superfamily. The TRPV1 channel is activated by different stimuli, including a pungent hot chili pepper component (capsaicin); on the other hand, the TRPM2 channel is activated by ADP-ribose (ADPR)^8–10^. In addition, the channel activities of TRPM2 and TRPV1 are affected by oxidative stress (independent of ADPR)^11,12^ and low pH (<5.9)^12,13^. The dorsal root ganglion neuron (DRGN) and sciatic nerve neuron (SciN) are important neurons for the induction of peripheral pain and FM^6,14^. The TRPM2 and TRPV1 channels are mainly expressed in the DRGN and SciN^8,10^. Hence, they are also involved in pain signaling and may be altered in chronic pain development. The results of our recent studies indicated that the TRPM2 and TRPV1 channels are activated in the DRGN and SciN by oxidative stress^15,16^. Therefore, oxidative stress and the acid-dependent activation of the TRPM2 and TRPV1 channels may be involved in the etiology of FM in the FM rat model.

It has been suggested that the mitochondria and oxidative stress may be important factors in the pathogenesis of FM^2,17–19^. However, there are also conflicting studies on mitochondrial oxidative stress in patients^20^. The essential trace element selenium (Se) acts as a regulator of the physiological functions of the nervous system, such as signal transduction and development^21^. Se also acts as a cofactor of the glutathione peroxidase (GSH-Px) enzyme and is incorporated into selenoproteins involved in antioxidant defenses^22^. Furthermore, Se is implicated as a neuroprotective agent in peripheral pain through the inhibition of apoptosis and regulation of the TRPM2 and TRPV1 channels^15,23,24^. Studies have reported a decreased blood Se level in patients with FM^25,26^. Furthermore, GSH-Px activity has been found to be drastically reduced in patients with FM^17–19,27^. Se may modulate Ca^2+^ influx via the TRPM2 and TRPV1 channels; thus, it may affect oxidative stress and apoptosis in the DRGN and SciN of rats with FM. This effect should be clarified in the DRGN and SciN of FM-induced rats.

The mechanism underlying DRGN and SciN injury through FM induction is still poorly understood. To our knowledge, there is no report of the effect of Se on apoptosis, oxidative stress, and Ca^2+^ entry in the DRGN and SciN of FM-induced rats. The aim of the current study is to determine the molecular mechanism of the effect of Se on apoptosis, oxidative stress, and Ca^2+^ entry through TRPV1 and TRPM2 regulation in the DRGN and SciN after FM induction.

## Results

### Effects of Se on mechanical hyperalgesia in FM-induced rats

**Figure 1:**
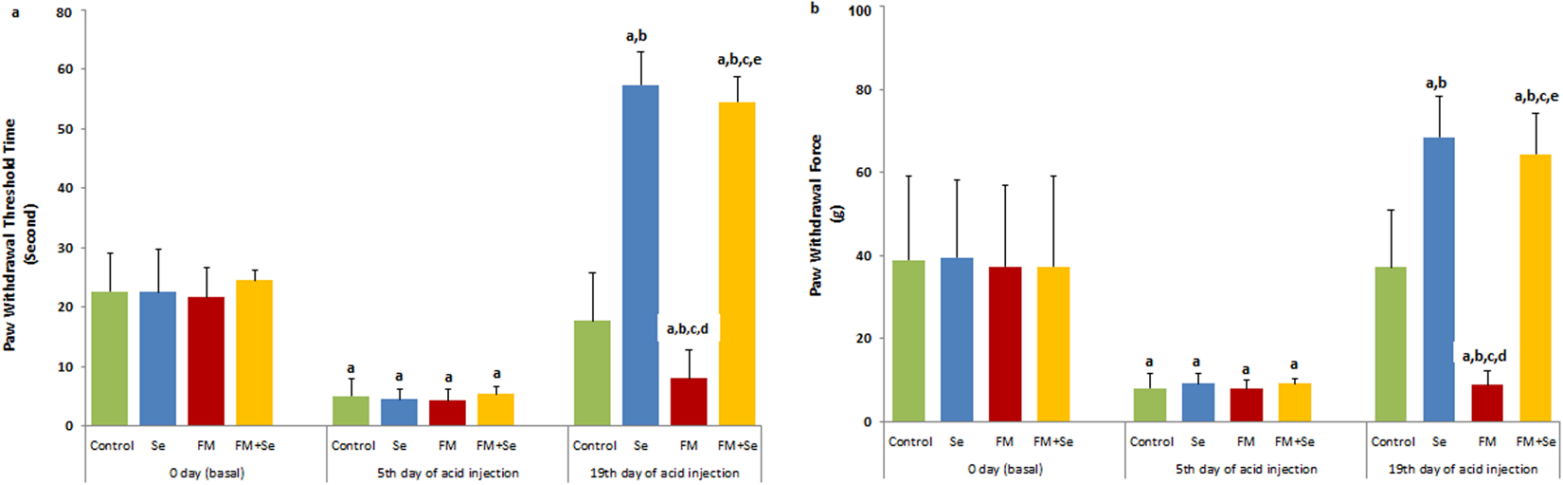
Effect of selenium (Se) treatment on paw withdraw threshold of Hot Plate Test (**a**) and paw withdrawal force of Von Frey test (**b**) in rats with FM. (n = 8–10 and mean ± SD). (^a^ *p* ≤ 0.001 versus basal groups. ^b^ *p* ≤ 0.001 5^th^ day groups. ^c^ *p* ≤ 0.001 versus control group of 19^th^ day group. ^*d*^ *p* ≤ 0.001 versus Se group of 19^th^ day group. ^e^ *p* ≤ 0.001 versus FM group of 19^th^ day group).

Five days after the second injection of acidic saline into the left gastrocnemius muscle, there was a significant decrease in the mechanical withdrawal threshold of the paw bilaterally, which continued to gradually decrease until the 19^th^ day in all periods analyzed, as demonstrated by hot plate (Fig. 1a) and von Frey (Fig. 1b) hyperalgesia assessments. On the other hand, compared with the control group, acid injections produced mechanical hyper-responsiveness on day 5^th^ and 19^th^ (*p* ≤ 0.001). However, when only Se was administered, a potent antihyperalgesic effect was observed in the injected paw (*p* ≤ 0.001). A similar antihyperalgesic effect was also observed in the contralateral paw when Se was administered to rats with FM. However, pretreatment with Se caused a marked increase in the sensitivity threshold to mechanical stimuli (*p* ≤ 0.001).

### Effects of Se on [Ca^2+^]_i_ concentration through modulation of TRPV1 and TRPM2 activation in the DRGN and SciN of FM-induced rats

**Figure 2:**
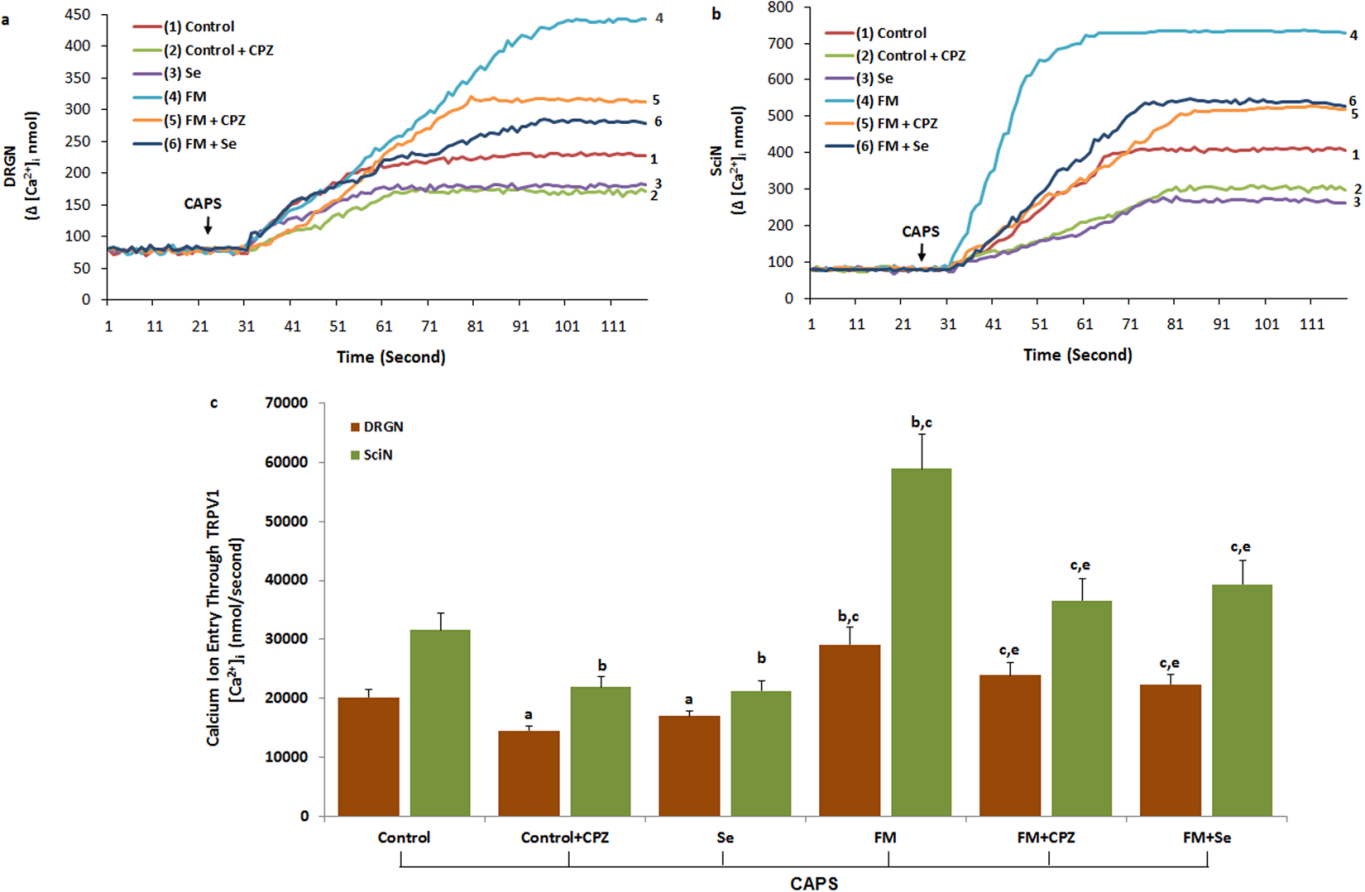
Effect of selenium (Se) treatment on [Ca^2+^]_i_ concentration through block of TRPV1 gate in dorsal root ganglion neuron (DRGN) (**a**) and sciatic nerve (SciN) (**b**) of control and FM-induced rats. (n = 8–10 and mean ± SD). The animals received intraperitoneal Se for 2 weeks after FM induction. Then, these dissected neurons of control and FM groups were further *in vitro* treated with CAPS (10 μM) and CPZ (0.1 mM) before loading Fura-2 for 120 seconds. (^a^ *p* ≤ 0.05 and ^b^ *p* ≤ 0.001 versus control. ^c^ *p* ≤ 0.001 and ^d^ *p* ≤ 0.05 versus control + CPZ and Se groups. ^e^ *p* ≤ 0.001 versus FM group (2c).

**Figure 3:**
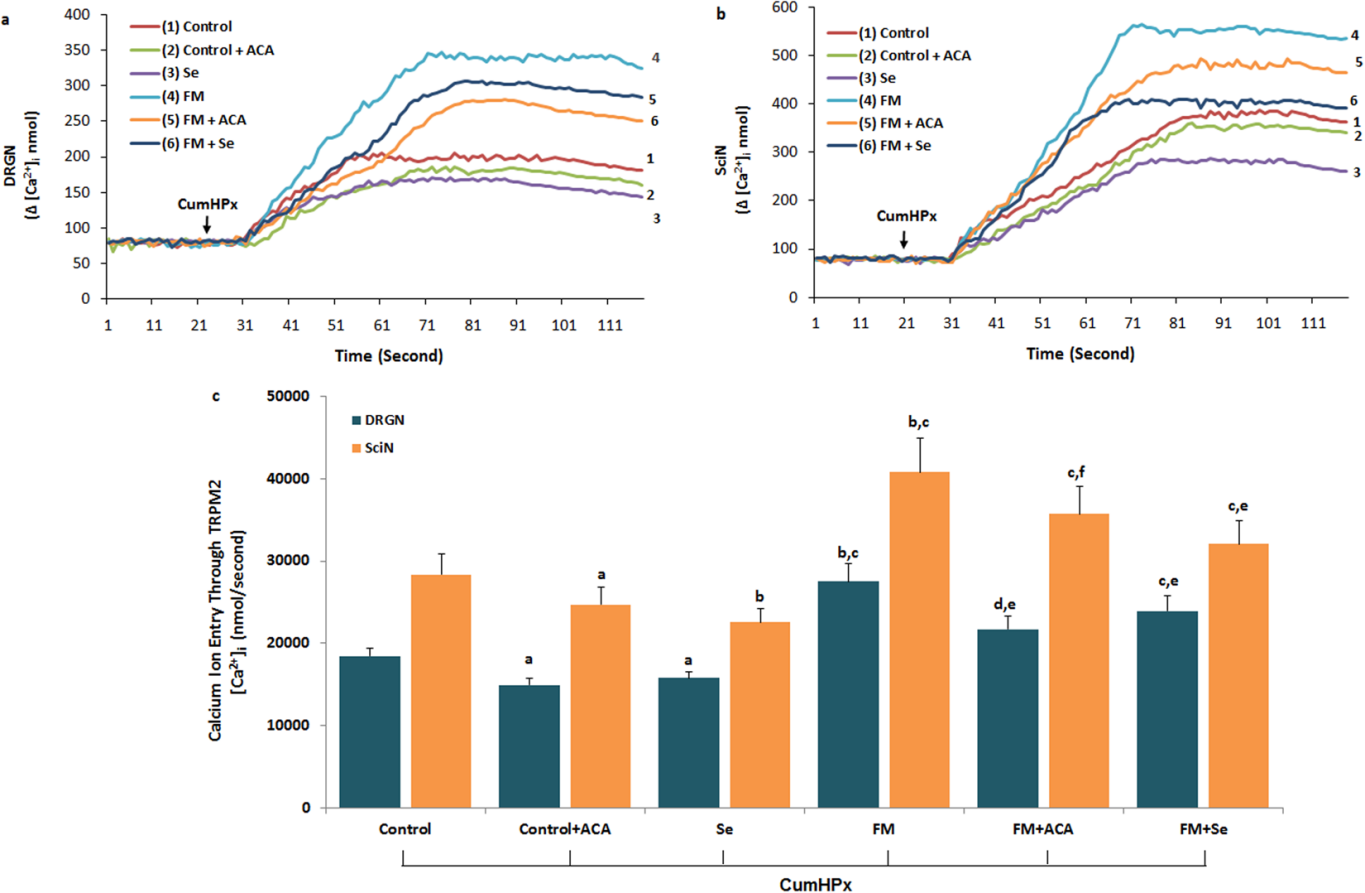
Effect of selenium (Se) treatment on [Ca^2+^]_i_ concentration through block of TRPM2 gate in dorsal root ganglion neuron (DRGN) (**a**) and sciatic nerve (SciN) (**b**) of control and FM-induced rats. (n = 8 and mean ± SD). The animals received intraperitoneal Se for 2 weeks after FM induction. Then, these dissected neurons of control and FM groups were further *in vitro* treated with CumHPx (1 mM) and ACA (0.025 mM) before loading Fura-2 for 120 seconds. (^a^ *p* ≤ 0.05 and ^b^ *p* ≤ 0.001 versus control. (^c^ *p* ≤ 0.001 and ^d^ *p* ≤ 0.05 versus control + ACA and Se groups. ^e^ *p* ≤ 0.001 versus FM group (3c).

To clarify the effect of Se on the TRPV1 and TRPM2 channels in the neurons, the neurons of Se-administrated rats were further gated by capsaicin (CAPS; 10 μM) and CumHPx (1 mM). Stimulations with CAPS and CumHPx caused a significant increase in [Ca^2+^]_i_ influx in the DRGN and SciN of FM-induced rats, which was attributed to the activation of Ca^2+^-permeable TRPV1 (Fig. 2a and b) and TRPM2 (Fig. 3a and b). As shown in Figs 2c and 3c, despite the higher concentration of [Ca^2+^]_i_ in the FM groups than in the control group, the TRPV1 antagonist (CPZ) and TRPM2 antagonist (ACA) could effectively decrease the concentration of [Ca^2+^]_I_, which was increased by experimental FM induction (*p* ≤ 0.001). The [Ca^2+^]_i_ concentration in the neurons was significantly (*p* ≤ 0.001) lower in the control + CPZ, control + ACA, FM + CPZ, and FM + ACA groups (*p* ≤ 0.001) than in the FM group. In addition, we found that the concentration of [Ca^2+^]_i_ was low in the neurons of the Se-treated group. The [Ca^2+^]_i_ concentration in the neurons was significantly lower in the FM + Se, FM + CPZ, and FM + ACA groups compared with the FM group (*p* ≤ 0.001). Therefore, Se could modulate the FM-induced [Ca^2+^]_i_ concentration by regulating TRPV1 and TRPM2 in the neurons.

### Effects of Se on CAPS-induced TRPV1 and ADPR-induced TRPM2 currents in the DRGN of control and FM-induced rats

**Figure 4:**
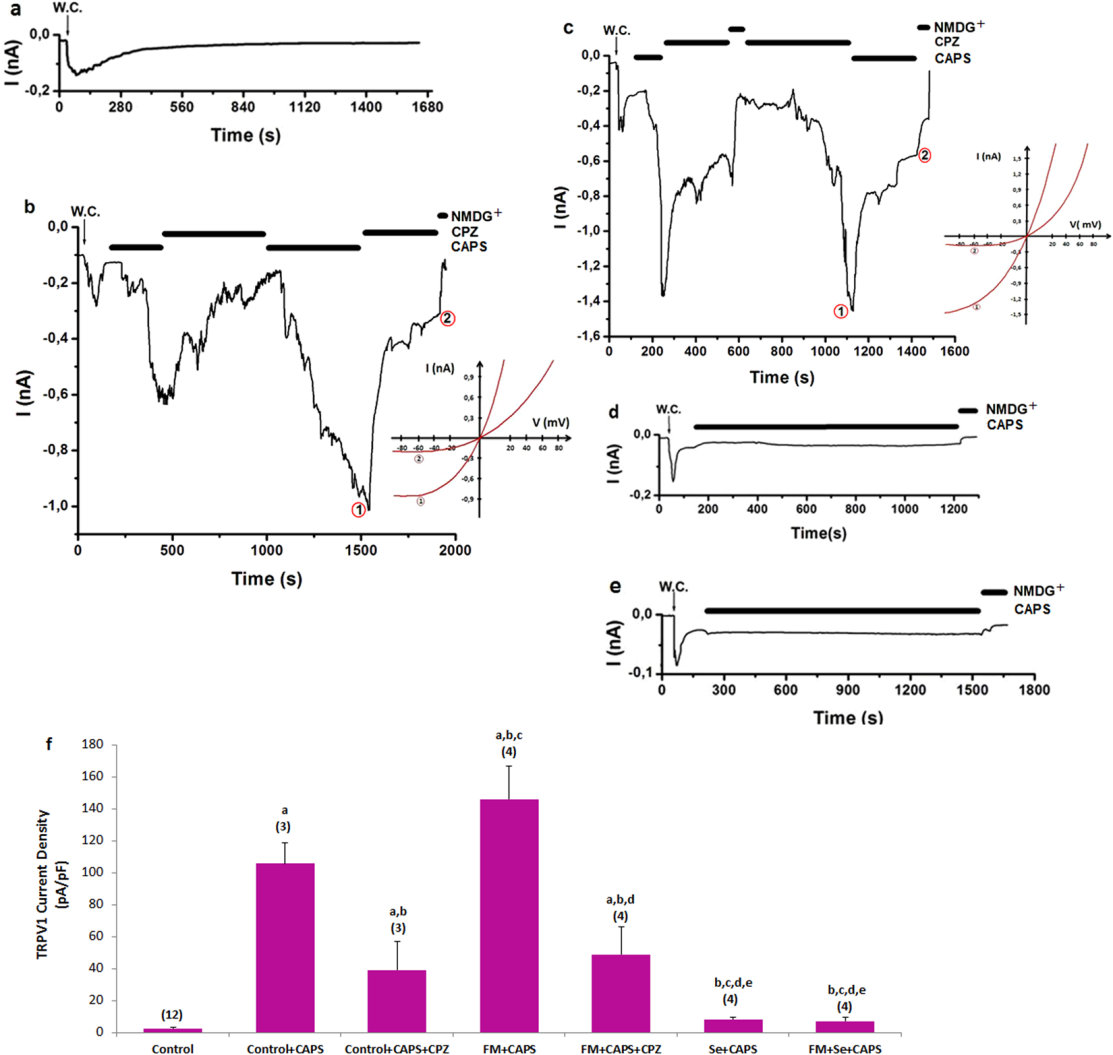
Effects of selenium (Se) on TRPV1 channel activation in dorsal root ganglion neuron (DRGN) of control and FM-induced rat. The TRPV1 currents in DRGN were stimulated by extracellular CAPS (10 μM in patch chamber) but they were blocked by extracellular CPZ (0.1 mM) in the patch-chamber. W.C. is whole cell. (**a**) Control: Original recordings from control neuron. (**b**) Control + CAPS group (without FM induction). (**c**) FM group (with FM induction). (**d**) FM + Se group: Se was administrated to the rats after FM induction. (**e**) Se group: Se was administrated to the rats without FM induction. (**f**) TRPV1 channel current densities in the DRGN. The numbers in parentheses indicated n numbers of groups. (^a^ *p* ≤ 0.001 versus control. ^b^ *p* ≤ 0.001 versus control + CAPS group. ^c^ *p* ≤ 0.001 versus control + CAPS + CPZ group. ^d^ *p* ≤ 0.001 versus FM + CAPS group. ^e^ *p* ≤ 0.001 versus FM + CAPS + CPZ group).

**Figure 5:**
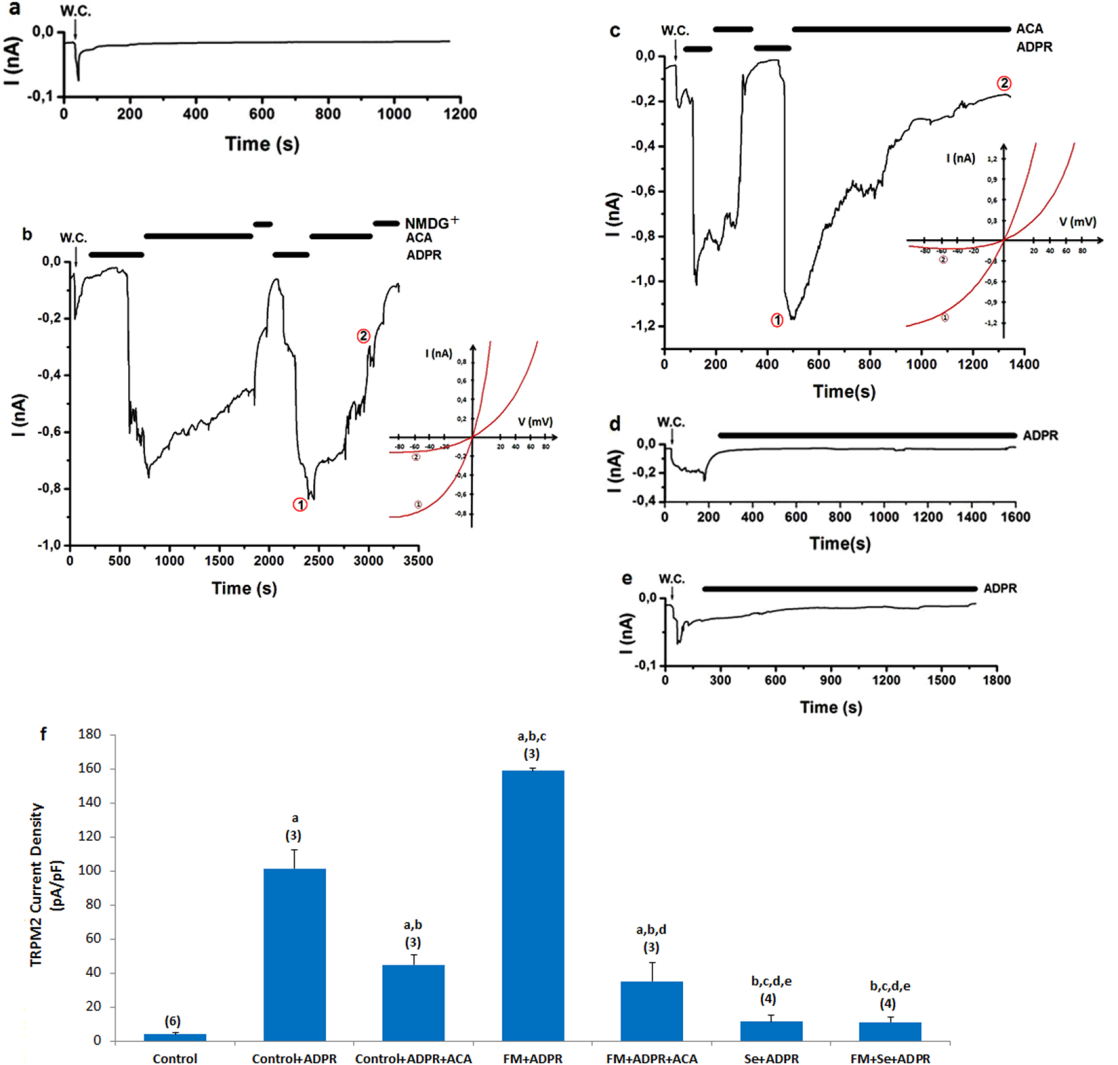
Effects of selenium (Se) on TRPM2 channel activation in dorsal root ganglion neuron (DRGN) of fibromyalgia (FM)-induced rat. The TRPM2 currents in DRGN were stimulated by intracellular ADPR (1 mM in patch pipette) but they were blocked by extracellular TRPM2 antagonist (ACA and 0.025 mM) in the patch-chamber. W.C. is whole cell. Control (without FM induction and stimulation): Original recordings from control neuron. (**b**) Control + ADPR group (without FM induction). (**c**) FM group (with FM induction). (**d**) FM + Se group: The rats received Se after FM induction. (**e**) Se group: The rats received Se without FM induction. (**f**) TRPM2 channel current densities in the DRGN. The numbers in parentheses indicated n numbers of groups were indicated by numbers in parentheses. (^a^ *p* ≤ 0.001 versus control. ^b^ *p* ≤ 0.001 versus control + ADPR group. ^c^ *p* ≤ 0.001 versus control + ADPR + ACA group. ^d^ *p* ≤ 0.001 versus FM + ADPR group. ^e^ *p* ≤ 0.001 versus FM + ADPR + ACA group).

The murine DRGNs in TRPV1 (Fig. 4b and c) and TRPM2 (Fig. 5b and c) experiments were gated by CAPS. CAPS- and ADPR-induced currents were reversibly blocked by CPZ, ACA, and NMDG^+^ (replacement of Na^+^), respectively. There were no currents in the absence of the agonists (CAPS and ADPR) (Figs 4a and 5a). The current densities in the neurons were significantly higher in the FM + CAPS and FM + ADPR groups compared with the control, control + CAPS, and control + ADPR groups (*p* ≤ 0.001); however, the current densities were significantly (*p* ≤ 0.001) lower in the control + CAPS + CPZ, control + ADPR + ACA, FM + CAPS + CPZ, and FM + ADPR + ACA groups than in the FM group (Figs 4f and 5f). The current densities in the neurons were decreased by Se treatments, and they were low in the Se + CAPS, Se + ADPR, FM + Se + CAPS, and FM + Se + ADPR groups (*p* ≤ 0.001). These results clearly indicated that CAPS and ADPR induced Ca^2+^ entry overload through the TRPV1 and TRPM2 channels. However, the FM-induced TRPV1 and TRPM2 currents through oxidative stress modulation were decreased by treatment with the antioxidant Se.

### Effect of Se on apoptosis, cell viability (MTT), caspase activity, intracellular ROS production, and JC-1 level in the SciN and DRGN of control and FM groups

**Figure 6:**
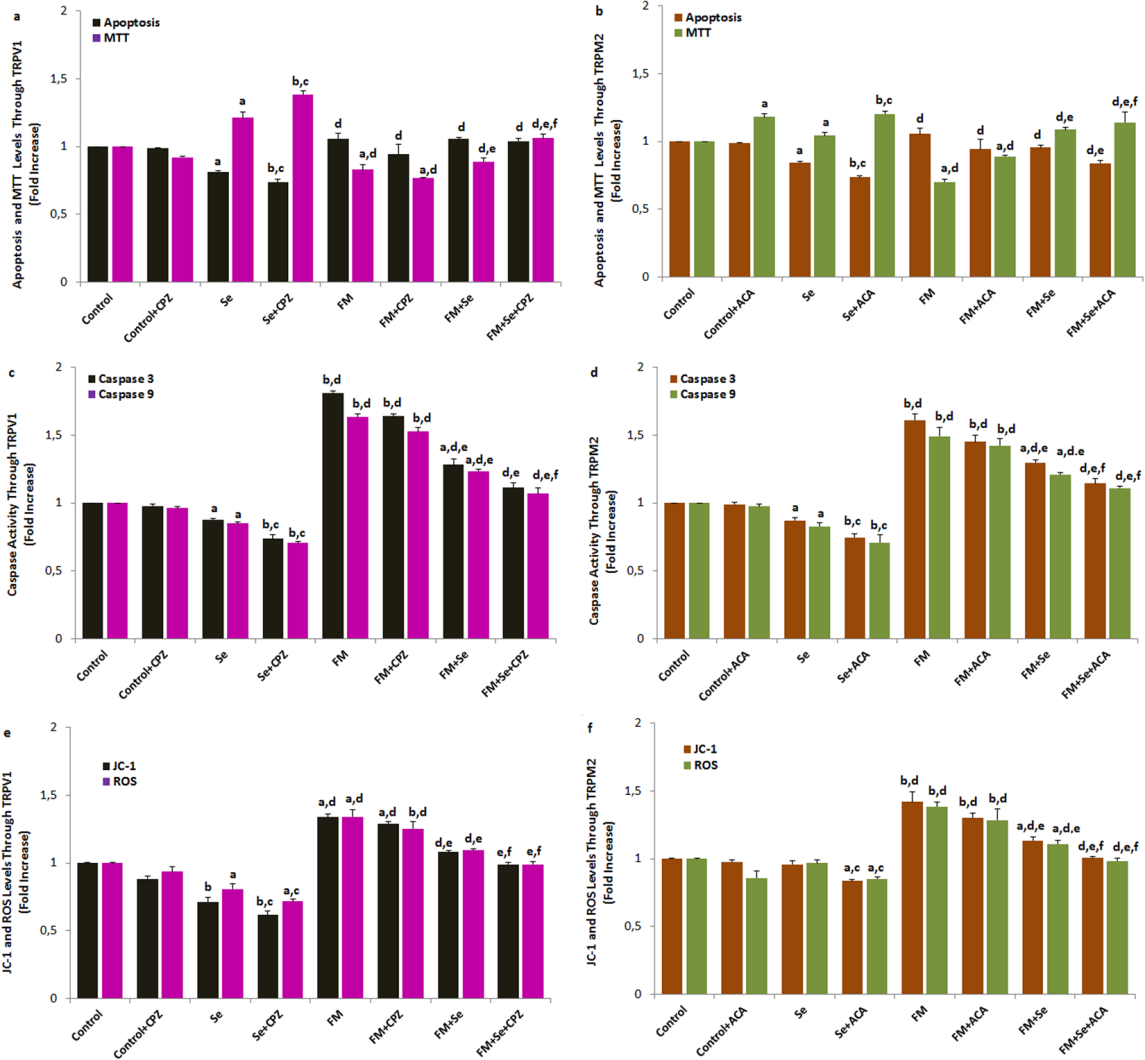
Effects of selenium on the apoptosis, cell viability (MTT), mitochondrial membrane depolarization (JC-1), intracellular ROS production and caspase 3 and 9 values through TRPV1 (**a**,**c** and **e**) and TRPM2 (**b**,**d** and **f**) channel activations in the DRGN of FM-induced rats (mean ± SD and n = 3). Values expressed as fold increase (experimental/control). These neurons were dissected from control, FM and treated animals. The TRPV1 and TRPM2 gates in the neurons were opened with capsaicin (10 μM) and cumene hydroperoxide (1 mM) although they were blocked by CPZ (0.1 mM) and ACA (0.025 mM), respectively. (^a^ *p* ≤ 0.05 and ^b^ *p* ≤ 0.001 versus control and control + CPZ groups. ^c^ *p* ≤ 0.05 versus Se group. ^d^ *p* ≤ 0.001 versus Se and Se + CPZ groups. ^e^ *p* ≤ 0.001 versus FM and FM + CPZ groups. ^f^ *p* ≤ 0.05 versus FM + Se groups).

Apoptosis levels (6a and b), caspase 3 and caspase 9 activities (6c and d), and JC-1 and ROS levels (6e and f) were markedly increased (*p* ≤ 0.001) following TRPV1 and TRPM2 stimulation in the DRGN (Fig. 6) and SciN (data are not shown) by FM induction; however, MTT levels were markedly decreased (*p* ≤ 0.001) in the neurons. The pre-treatment of cells with CPZ (without Se) followed by CAPS stimulation reversed CAPS- and CumHPx-induced oxidative cytotoxicity (apoptosis, MTT, JC1-1, ROS, caspase 3, and caspase 9 values), and the CPZ and ACA treatment resulted in significant additive effects with a 20–50% increase in cell viability (Fig. 6a and b). In addition, apoptosis, JC-1, and ROS levels and caspase 3 and caspase 9 activities were markedly decreased (*p* ≤ 0.05 and *p* ≤ 0.001, respectively) in the neurons of the FM groups following the blockage of the TRPV1 and TRPM2 channels by Se with/without CPZ and ACA treatments; however, MTT levels were markedly increased (*p* ≤ 0.05 and *p* ≤ 0.001, respectively) in the FM groups by Se with/without CPZ and ACA treatments. These results implied that TRPV1 and TRPV1 cytotoxicity may be attributed to TRPV1 and TRPM2 activation.

### Effect of Se on caspase 3, caspase 9, and PARP1 expression level in the DRGN, SciN, and muscle of FM-induced rats

**Figure 7:**
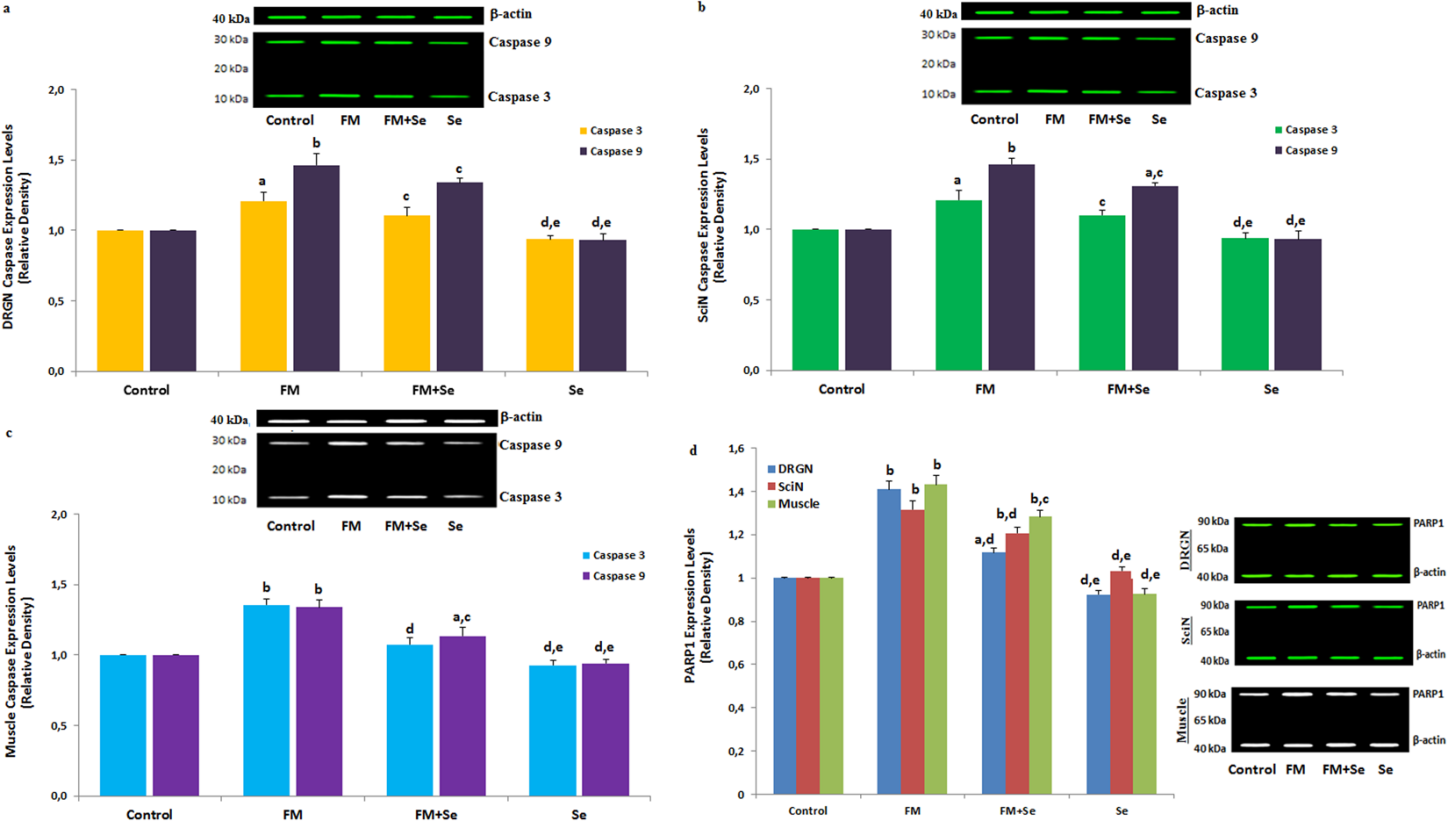
Effects of selenium on the caspase 3, caspase 9 and PARP1 expression levels in DRGN (**a** and **d**), SciN (**b** and **d**) and gastrocnemius muscle (**c** and **d**) of rats with FM (mean ± SD and n = 3). (^a^ *p* ≤ 0.05 and ^b^p ≤ 0.001 versus control. ^c^ *p* ≤ 0.05 and ^d^ *p* ≤ 0.001 versus FM group. ^e^ *p* ≤ 0.001 versus FM + Se group).

PARP1 activity plays an essential role in DNA damage, and the TRPM2 channel agonist ADPR is produced in the nucleus by stimulation of oxidative stress. In apoptotic pathways, caspase 8 activates a proteolytic caspase cascade that transmits and amplifies death signals by the activation of apoptotic caspases such as executioner caspase 3 and initiator 9. These initiator and executioner caspases cleave several substrate proteins including PARP1, resulting in the self-destruction of the cells^28^. Caspase 3 and caspase 9 expression levels are associated with the progress of apoptosis in neuronal injuries^15,24^. In the current study, the expression levels of caspase 3, caspase 9, and PARP1 in the DRGN (Fig. 7a and d), SciN (Fig. 7b and d), and muscle (Fig. 7c and d) were markedly higher (*p* ≤ 0.001) in FM group than in the control group. However, the expression levels of caspase and PARP1 in the three samples were decreased by FM + Se treatments, and their expression levels in the SciN (*p* ≤ 0.05), DRGN (*p* ≤ 0.05), and muscle (*p* ≤ 0.001) were significantly lower in the FM + Se group than in the FM group. In addition, the expression levels of caspase and PARP1 in the three samples were further decreased by Se treatments. These results demonstrated of the roles of caspase 3 and 9 in the apoptotic pathway activated by FM; these caspases could trigger a proteolytic cascade that amplifies death signals by inducing DNA damage, executioner caspase 3, and initiator 9.

### Effects of Se on MDA, GSH, GSH-Px, and antioxidant vitamin concentration

Decreased blood GSH, GSH-Px, and antioxidant vitamin levels were reported in patients with FM^17–19^. On the other hand, an increase in their levels^17^ and a decrease in muscle pain intensity^29^ were reported in patients with FM following α-tocopherol supplementation. Furthermore, GSH-Px activity has been found to be drastically reduced in patients with FM^17^. Therefore, we measured MDA concentration, GSH concentration, and GSH-Px activity in the muscle, brain homogenate, and hemolyzed erythrocytes of rats with FM to clarify the role of oxidative stress in the etiology of FM (Table 1).

**Table 1:** Effects of selenium (Se) on lipid peroxidation (MDA), reduced glutathione (GSH) levels, glutathione peroxidase (GSH-Px) activity and antioxidant vitamin concentrations in DRGN, brain, erythrocyte, muscle and sciatic neurons (SciN) in fibromyalgia (FM)-induced rats (mean ± SD). ^a^ *p* ≤ 0.05, ^b^ *p* ≤ 0.01 and ^c^ *p* ≤ 0.001 versus control. ^d^ *p* ≤ 0.05 and ^e^ *p* ≤ 0.001 versus Se group. ^f^ *p* ≤ 0.05 and ^g^ *p* ≤ 0.001 versus FM group.

Values		Control (n = 8)	Se (n = 8)	FM (n = 10)	FM + Se (n = 10)
MDA (μmol/g pr)	DRGN	19.70 ± 0.85	18.60 ± 0.98^a^	21.70 ± 0.70^a,d^	19.40 ± 0.76^f^
	Brain	20.70 ± 1.11	19.40 ± 2.58	24.00 ± 2.07^a,d^	20.90 ± 1.14^f^
	Erythrocyte	22.20 ± 2.74	23.00 ± 2.16	24.20 ± 1.46	22.90 ± 2.04
	Muscle	17.10 ± 1.08	16.80 ± 1.46	18.80 ± 0.96^a,d^	22.90 ± 2.04
	Plasma (μmol/l)	2.04 ± 0.06	1.88 ± 0.10^a^	2.48 ± 0.16^a,e^	2.16 ± 0.12^g^
	SciN	21.40 ± 2.33	18.80 ± 2.13^a^	23.60 ± 2.61^b,e^	20.10 ± 0.97^d,g^
GSH (μmol/g pr)	DRGN	11.80 ± 0.70	12.20 ± 1.16	10.80 ± 0.71^a,d^	11.70 ± 1.04^f^
	Brain	16.80 ± 0.86	18.40 ± 0.79^b^	15.60 ± 0.36^b,e^	17.00 ± 0.61^d,g^
	Erythrocyte	15.70 ± 0.79	15.50 ± 0.64	15.40 ± 0.48	15.50 ± 0.52
	Muscle	13.00 ± 0.70	15.30 ± 0.97^c^	12.00 ± 0.37^c,e^	13.30 ± 0.52^e,f^
	SciN	13.40 ± 1.63	14.20 ± 0.75^a^	12.20 ± 1.15^c,e^	14.00 ± 0.67^g^
GSH-Px (μmol/g pr)	DRGN	21.51 ± 1.05	26.59 ± 1.85^b^	19.83 ± 2.87^a,e^	22.91 ± 2.71^e,f^
	Brain	23.40 ± 2.79	24.50 ± 3.03^a^	21.60 ± 2.77^a,e^	23.00 ± 2.44^d,f^
	Erythrocyte	24.27 ± 2.94	29.05 ± 1.09^c^	23.60 ± 1.87^a^	28.93 ± 2.19^c,e,g^
	Muscle	18.80 ± 1.20	20.20 ± 1.70^a^	15.70 ± 1.54^b,e^	17.60 ± 1.05^e,g^
	SciN	22.59 ± 1.45	23.76 ± 1.75^a^	20.37 ± 1.08^b,e^	22.49 ± 1.28^d,f^
Retinol (μmol/g tissue)	Brain	2.86 ± 0.38	2.91 ± 0.22	2.79 ± 0.23	2.97 ± 0.37
	Muscle	2.22 ± 0.26	2.15 ± 0.19	2.32 ± 0.17	2.26 ± 0.19
	Plasma (μmol/l)	2.93 ± 0.17	2.88 ± 0.27	2.82 ± 0.19	2.78 ± 0.23
α-Tocopherol (μmol/g tissue)	Brain	13.60 ± 0.47	15.40 ± 0.84^c^	12.50 ± 0.58^a,e^	14.30 ± 0.75^d,g^
	Muscle	10.60 ± 0.93	11.10 ± 0.45	9.19 ± 0.61^a,e^	10.30 ± 0.59^f^
	Plasma (μmol/l)	22.30 ± 1.14	26.30 ± 1.09^c^	22.20 ± 1.20^e^	23.70 ± 1.05^c^
β-Carotene (μmol/g tissue)	Brain	0.94 ± 0.10	1.07 ± 0.10	0.98 ± 0.12	1.01 ± 0.15
	Muscle	0.79 ± 0.17	0.81 ± 0.12	0.85 ± 0.10	0.88 ± 0.13

GSH level and GSH-Px activity in the DRGN, brain, muscle, erythrocyte, and SciN and α-tocopherol concentration in the brain, muscle, and plasma were significantly decreased (*p* ≤ 0.05 and *p* ≤ 0.001, respectively) by the induction of FM; however, MDA levels in the samples were markedly decreased (*p* ≤ 0.05 and *p* ≤ 0.001, respectively) (Table 1). On the other hand, GSH-Px activity and GSH level (in the DRGN, brain, muscle and SciN) and α-tocopherol concentration (in the brain, muscle, and plasma) were increased in the FM group following Se treatment (*p* ≤ 0.05 and *p* ≤ 0.001, respectively). There was no change in retinol and β-carotene concentration in the four groups (Table 1).

## Discussion

The results of the current study indicated that Se treatment might decrease FM-induced pain intensity, [Ca^2+^]_i_ accumulation, mitochondrial ROS, and apoptosis levels in the DRGN and SciN by blocking the TRPM2 and TRPV1 channels. To our knowledge, this is the first evidence demonstrating the FM pathophysiological process and implicating the DRGN and SciN in central and peripheral pain diseases.

TRPV1 is a pronociceptive polymodal receptor that senses CAPS, oxidative stress, high temperature, and acidic pH. Oxidative stress and acidic pH play significant roles in the activation of TRPM2 and TRPV^1,12,13^. The activation of TRPM2 and TRPV1 through oxidative stress and acidic pH could enhance [Ca^2+^]_i_ accumulation, and they are involved in several physiological and pathological processes such as neuronal apoptosis and neuronal recovering signaling^5,15,30^. Therefore, TRPM2 and TRPV1 could act as the final oxidative stress mediators that function via distinct intracellular signaling pathways such as NADPH oxidase^31^. The blockage of TRPM2 and TRPV1 generally results in antinociceptive effects in animal models; however, the roles of TRPM2 and TRPV1 in maintaining acid- and oxidative stress-induced chronic hyperalgesia in animal models have not been elucidated. Recent studies have revealed that TRPV1 may play a role in the development of hyperalgesia after the induction of acid-induced FM^5^. Se as a cofactor of the cytosolic antioxidant enzyme GSH-Px is a strong antioxidant^21,22^, and it has strong superoxide radical scavenger and NADPH oxidase blocker effects in several types of cells^5,32^. In recent studies, we observed that Se treatment decreased the levels of Ca^2+^ influx by blocking TRPM2 and TRPV1 in the DRGN and SciN of rats^15,30^. To our knowledge, there is no report on calcium signaling and pain intensity in relation to the activation of TRPM2 and TRPV1 in the DRGN and SciN of rats with FM. In the current study, we observed that pain intensity and Ca^2+^ influx through the TRPM2 and TRPV1 channels were increased in the DRGN and SciN of FM-induced rats; however, pain intensity and Ca^2+^ influx were decreased in the neurons following treatment with the antioxidant Se. Similar to the pain intensity and Ca^2+^ results of the current study, decreased chronic hyperalgesia has been demonstrated by blocking CaV3.2 T-type Ca^2+^ channel signaling in mice at 15 min before the second acid injection in another study^33^. In addition, TRPV1 overexpression in the development of a FM-like pain state has been reported in an acid-injected TRPV1-null mice model of FM^34^.

The increased activation of cation channels including TRPM2 and TRPV1 leads to a high [Ca^2+^]_i_ concentration, which disrupts the Ca^2+^ contents of the intermembrane space through mitochondrial permeability transition activation^35^. The dysfunction of the mitochondria activates three pathways: (1) generation of endogenous mitochondrial ROS, (2) apoptosis through caspase production, and (3) DNA damage through PARP1 activation^34^. The role of TRPM2 and TRPV1 in mitochondrial dysfunction and apoptosis in the DRGN and SciN has been reported in recent studies^15,34^. In the current study, apoptosis, caspase 3, caspase 9, PARP1, JC-1, and intracellular ROS values were increased in the SciN and DRGN following FM induction; however, their values were decreased in the neurons by blocking TRPM2 and TRPV1 with Se.

The antagonist effects of Se on the TRPM2 and TRPV1 channels in the DRGN were revealed in recent studies^15,24^. In addition, the involvement of NADPH oxidase and protein kinase C molecular pathways in the activation of TRPM2 and TRPV1 in the DRNG were reported in a recent study^31^. Besides functioning as an antagonist of TRPM2 and TRPV1, Se also inhibits NADPH oxidase in various cell lines^32,33^. Based on present data on Se, we hypothesize that Se as a potential antagonist of TRPM2 and TRPV1 may also function via this mechanism. Indeed, we found that treatment with Se resulted in decreased FM-induced mitochondrial ROS in the DRGN and SciN, and FM-induced apoptosis was reversed by Se treatments. Therefore, the current results indicated that Se may modulate FM-induced cellular oxidative stress in the DRGN and SciN of rats with FM. It is well known that patients with FM are under oxidative stress because of the ischemia of tender points and abnormalities in mitochondrial functions, resulting in a delicate balance between blood ROS levels and the antioxidant capabilities of the cell^17–19,36^. Therefore, increases in ROS above the basal level could disrupt this fine balance, thereby triggering ROS-induced apoptosis. This may explain why Se was able to inhibit ROS, apoptosis, caspase 3, and caspase 9 in the DRGN and SciN of rats with FM.

The human body is equipped with a complete arsenal of defenses against ROS. These ROS are scavenged by enzymes, such as GSH-Px and catalase, and non-enzymatic species, such as GSH and vitamin E^37^. α-Tocopherol is the major scavenger of ROS in the lipid phase of neuronal membranes, and its oxidized form is recovered by the antioxidant action of GSH. An imbalance between the oxidant and antioxidant system is a common feature in the clinical dysfunction of the nervous system. It is well known that an increase in mitochondrial ROS and a decrease in GSH-Px activity and Se level are known to be implicated in the etiology of pain and FM^17–19,25–27^. In addition to acting as a cofactor of GSH-Px, Se is an important antioxidant enzyme in the brain and neurons for removing lipid hydroperoxides and hydrogen peroxide^21^. In the current study, we observed that GSH-Px activity, GSH concentration, and α-tocopherol concentration were reduced in the DRGN, SciN, brain, muscle, and plasma; however, they were increased to near basal levels following Se treatments. These results demonstrated that Se administration conferred protection against FM in the rats, possibly by limiting the lifetime of ROS. Retinol and β-carotene concentration in the brain and muscle did not change in the four groups; however, they also have antioxidant roles in neurons. The adaptive antioxidant responses of retinol and β-carotene were accompanied by GSH-Px activity, GSH concentration, and α-tocopherol concentration upregulation.

In summary, our data suggested for the first time that the mechanisms of FM may be mediated via the TRPM2 and TRPV1 channels by inducing mitochondrial ROS, apoptosis, and pain in the DRGN and SciN. However, the pain, oxidant, and apoptotic effects of FM were reversed through the blockage of TRPM2 and TRPV1 in the neurons by Se treatment. Therefore, the use of Se may be an effective novel approach for treating FM-induced pain, mitochondrial oxidative stress, and apoptosis. In addition, the TRPM2 and TRPV1 channels may be important pharmacological targets in the treatment of FM-induced apoptosis and pain.

## Methods

### Animals

It is well known that incidence rate of FM is high in women^1–3^. Hence, we used 36 female Wistar rats (aged 12 weeks old and 170 ± 10 g body weight) in the current study. The rats were housed in controlled-temperature (21 ± 2 °C) and humidity (65%) rooms, under a 12:12 h light-dark cycle, with *ad libitum* access to water and food until use. Hot plate and Von Frey tests were performed between 9:00 and 10.00 a.m. at baseline, 5^th^ and 19^th^ days of the experiments.

### Availability of data and materials

Data and approve of rats were taken from Experimental Animal Research Center of Suleyman Demirel University (SDU) according to protocol number (HADYEK-21438139-320). All methods in the manuscript were performed in accordance with the relevant guidelines and regulations of SDU by including a statement in the methods section to this effect. The dataset and analyses were generated in Neuroscience Research Center of SDU and they are available from the corresponding author on reasonable request. Graphics and tables in the manuscript were prepared by the corresponding author.

### Study groups

The rats were divided into four groups as follows: The control group (n = 8) had no FM and was not administrated. They received intraperitoneal (i.p.) 0.9% w/v saline solution for 2 weeks. In the Se group (n = 8), they received i.p. Se 1.5 mg/kg/over day for two weeks (total seven doses)^15^. In FM group (n = 10), the rats were exposed FM induction procedure^4^. Then, the rats received i.p. saline solution for 2 weeks. In FM + Se group (n = 10), the rats received Se (same as the Se group) after FM induction (same as the FM group).

Twelve hours after the last saline solution and Se dose administration, all rats were decapitated under propofol inhalation anesthesia in accordance with SDU experimental animal legislation. Selenium (Sodium selenite, Sigma Chemical Co., MO, USA) was diluted to appropriate concentration in sterile 0.9% w/v saline solution. In patch-clamp experiment and [Ca^2+^]_i_ concentration assays, the DRGN and SciN were further treated with CumHPx (1 mM) or ADPR (1 mM) and capsaicin (10 μM) for activation of TRPM2 and TRPV1 channels, respectively and they were also blocked the TRPM2 channels antagonist, N-(p-amylcinnamoyl) anthranilic acid (ACA and 0.025 mM) and TRPV1 antagonist, capsazepine (CPZ and 0.1 mM). Doses of capsaicin and CPZ on TRPV1 in cells are changing between 100 nM and 0.1 mM. In a recent study^31^, we tested different doses of capsaicin and CPZ in the DRGN and TRPV1 against and antagonist doses of capsaicin and CPZ in the study was found as 10 μM and 0.1 mM, respectively. Therefore, the doses were used in the current study.

### Induction of hyperalgesia

For produce a bilateral long-lasting hyperalgesia similar to fibromyalgia in humans, acidic saline solution (adjusted to pH 4.0) was injected into the gastrocnemius muscle in the rats^4^. Before the first injection of acidic or normal saline into the gastrocnemius muscle, paw withdrawal threshold values of the rats were recorded in order to record the baseline value. Each animal received two repeated injections of the acidic saline solution (100 μl) in the same unilateral gastrocnemius muscle under propofol inhaled anesthesia, a procedure which is repeated five days later^4^. After the second injection of the acidic saline, hyperalgesia in the animal is detected increase the paw withdrawal threshold by the von Frey filament and hot plate tests in the muscle.

### Hyperalgesia tests

The assessment of hyperalgesia was measured by the using calibrated von Frey filaments (20PC Aesthe Model, NO. 160615, Muromachi Kikai Co., Ltd. Tokyo, Japan), to the plantar aspect of the hind paw of mice that were kept in suspended wire mesh cages. A response was indicated by lifting of the hind paw^38^.

Heat-controlled plate (Varioma, Thermo Fischer Inc., Langenselbold, Germany) for Hot Plate test was used to assess paw withdrawal latency to thermal nociceptive stimuli. The hot plate test apparatus consisted of an electrically heated surface kept at a constant temperature of 55.0 ± 0.6 °C. Each mouse was placed on hot plate, and the reaction time was measured until the mouse either demonstrated hind paw licking or jumping (‘up and down’ method). A cutoff time of 60 s was used to prevent tissue damage for hot plate test^38^. Withdrawal threshold was determined by sequentially increasing and decreasing the stimulus strength.

### Preparation of brain, blood, primary DRGN and SciN samples

Details of isolations of DRGN and SciN were given in previous studies^15,34^. Briefly, the DRGN and SciN were minced with iridectomy scissors and incubated with enzymes including trysin (type III, Sigma) and 0.5 mg/ml collagenase (type XI, Sigma) in 5 ml DMEM at 37 °C in a shaking bath for 40 min after removing the attached nerves and surrounding connective tissues. To stop the enzymatic digestion 1.25 mg/ml soybean trypsin inhibitor (type II-S1, Sigma) was added. After dissociation with a sterile syringe, the DRGN and SciN suspensions of mediums were centrifuged at 1,500 g and the medium and high size neurons were removed for the analysis. The isolated neurons were transferred into a 35-mm culture dish and kept still for at least 30 min.

The brain was also taken as follows; the cortex was dissected out after the brain was split in the mid-sagittal plane. The gastrocnemius muscle samples from right legs were also taken. After preparing brain and muscle homogenates in ice-cold Tris-HCl buffer (50 mM, pH 7.4), they were stored at −85 °C for analyses of lipid peroxidation and antioxidants. Half of the muscle sample with DRGN and SciN was frozen at −85 °C for using Western blot analyses. Plasma and erythrocyte samples from anticoagulated blood (sodium EDTA) were obtained as described in a previous studies^17,18^ and they were stored at −85 °C.

### Electrophysiology

Whole-cell voltage clamp recording was taken from the DRGN at 22–24 °C (EPC10 patch-clamp set, HEKA, Lamprecht, Germany). Resistances of the recording electrodes were adjusted to about 3–6 MΩ by a puller (PC-10 Narishige International Limited, London, UK). We used standard extracellular bath and pipette solutions as described in previous studies^16,30^. Holding potential of the patch-clamp analyses in the DRGNs was −60 mV. Voltage clamp technique was used in the analyses and current-voltage (I–V) relationships were obtained from voltage ramps from −90 to +60 mV applied over 200 milliseconds. All experiments were performed at room temperature (22 ± 1 °C).

In the experiments, TRPM2 is intracellularly (via patch pipette) gated by ADPR (1 mM), although they were extracellularly (via patch chamber) blocked by ACA (0.025 mM). TRPV1 was extracellularly gated by CAPS (10 μM), and the channels were extracellularly blocked by CPZ (0.1 mM). For the analysis, the maximal current amplitudes (pA) in a DRGN were divided by the cell capacitance (pF), a measure of the cell surface. The results in the patch clamp experiments are the current density (pA/pF).

### Measurement of [Ca^2+^]_i_ concentration in DRGN and sciatic nerve

The DGRN and SciN samples were separately seeded in sterile cell culture flasks. Then they were loaded with 4 μM Fura-2/AM in loading buffer for 45 min at 37 °C in the dark, washed twice with the phosphate buffer, incubated for an additional 30 min at 37 °C to complete probe de-esterification. The groups were exposed to the stimulations in a water-jacketed cuvette (37 °C) with continuous magnetic stirring. Fluorescence was detected by using a Carry Eclipse Spectrofluorometer (Varian Inc, Sydney, Australia). The fluorescence at 505 nm was measured at 1 second intervals after excitation at 340 nm and 380 nm, respectively.

For calibration of [Ca^2+^]_i_, maximum and minimum fluorescence values were obtained by adding the detergent Triton X-100 (0.1%) and the Ca^2+^ chelator EGTA (10 mM) sequentially at the end of each experiment. Calculation of the [Ca^2+^]_i_ concentrations were described in previous studies^39,40^, assuming a Kd of 155 nM. The [Ca^2+^]_i_ concentrations in TRPM2 and TRPV1 experiments were recorded by using the integral of the rise in [Ca^2+^]_i_ for 120 seconds after addition of CumHPx (1 mM) or capsaicin (10 μM), respectively. The [Ca^2+^]_i_ concentration is expressed as nanomolar (nM) taking a sample every second as previously described^39,41^.

### Measurement of intracellular ROS production and mitochondrial membrane depolarization

Dihydrorhodamine (DHR)123 is an uncharged and nonfluorescent intracellular ROS production indicator and the level of intracellular ROS was assessed by fluorescence microplate reader (Infinite pro200, Tecan Life, Männedorf, Switzerland) with DHR123, which oxidizes to cationic rhodamine 123 in the presence of ROS, as described previously^39^. The resulting data were normalized using the control values. 5,5′,6,6′-tetrachloro-1,1′,3,3′-tetraethylbenzimidazolocarbocyanine iodide (JC-1) dye was used for measurement of the mitochondrial membrane potential and it was determined in he a plate reader (Infinite pro200) by using JC-1 as described in previous studies^40,42^. The ROS and JC-1 values were expressed as fold-increase over the pretreatment level after calculating fluorescence units/mg protein.

### Assay for apoptosis, cell viability, and caspase 3 and 9 values

The apoptosis levels were determined in a spectrophotometer (UV-1800, Shimadzu, Kyoto, Japan) by using a commercial kit of Biocolor Ltd. (Northern Ireland) as described in a previous study^41^. The method is based on loss of asymmetry in membranes of apoptotic neurons. We used to cell viability analyses as 3-(4,5-Dimethylthiazol-2-yl)-2,5-diphenyltetrazolium bromide (MTT) in the neurons as described elsewhere^41^ and absorbance values of MTT were recorded by the spectrophotometer (UV-1800) at 490 nm.

The determinations of caspase 3 and caspase 9 activities in the sciatic nerve and DRGN neurons were performed in the microplate reader (Infinite pro200) by using caspase 3 (N-acetyl-Leu-Glu-His-Asp-7-amino-4-methylcoumarin (AC-LEHD-AMC), Bachem, Bubendorf, Switzerland) and caspase 9 (N-acetyl-Asp-Glu-Val-Asp-7-amino-4-methylcoumarin (ACDEVD-AMC), Sigma) substrates. Details of the assays were indicated in recent studies^16,34^. The apoptosis, MTT, caspase and 9 values were expressed as fold-increase after calculating fluorescence units/mg protein.

### Lipid peroxidation, GSH-Px, reduced glutathione (GSH) and antioxidant vitamin analyses in plasma, erythrocytes, muscle (gastrocnemius) and brain

The GSH concentration and GSH-Px activity in the DRGN, SciN, muscle, brain homogenate and hemolyzed erythrocyte were spectrophotometrically (UV-1800) assayed at 412 nm by using the method of Sedlak and Lindsay^43^ and Lawrence and Burk^22^, respectively. Lipid peroxidation levels as malondialdehyde (MDA) in the DRGN, SciN, muscle, brain homogenate, plasma and hemolyzed erythrocyte were measured with the thiobarbituric-acid reaction by using method of Placer *et al*.^44^. The GSH-Px activity, GSH and MDA concentrations were expressed as international unit (IU) of GSH oxidized/min/μg protein (IU/μg pr) and μmol/g protein (μmol/μg pr), respectively. Method of Bradford was used in the muscle and brain homogenate, and hemolyzed erythrocytes for the determination of protein contents.

Results of recent studies indicated low vitamin A (retinol), vitamin E (α-tocopherol) and β-carotene concentrations in plasma of patients with FM. For further clarifying role of antioxidant vitamins in FM-induced oxidative stress, retinol, β-carotene and α-tocopherol were spectrophotometrically determined in the brain, gastrocnemius muscle and plasma samples by a modification of the method described by Suzuki and Katoh^45^ and Desai^46^. Calibration was performed using standard solutions of all-trans retinol, β-carotene and α-tocopherol in hexane. All antioxidant vitamin values in plasma, muscle and brain were expressed as μmol/l for plasma and μmol/g tissue for brain and muscle, respectively.

### Western Blot analyses

Standard procedures are used in the Western Blot analyses of gastrocnemius muscle, SciN and DRGN^16,34^. Samples were homogenized in ice-cold RIPA buffer. The protein concentration in the supernatant was determined using the Bradford’s method. Membrane blots were incubated overnight at 4 °C with the following primary antibodies: caspase 3 (p17-specific Polyclonal Antibody), caspase 9 (p35/p10 Polyclonal Antibody), β actin (polyclonal antibody), poly-ADPR polymerase 1 (PARP1) (polyclonal antibody). The primary antibodies were purchased from Proteintech (Istanbul, Turkey) although secondary antibodies (Rabbit IgG, HRP-linked from donkey) were purchased from GE Healthcare (Amersham, UK). Relative levels of immunoreactivity in ECL Western HRP Substrate (Millipore Luminate Forte, USA) were quantified using Syngene G:Box Gel Imagination System (UK). Rabbit anti-β-actin (1:2000) was used as an internal control for the concentration of proteins loaded. Obtained values were expressed as relative density over the control level.

### Statistical analyses

All data were represented as means ± standard deviation (SD). The data were analyzed by using 17.0 version of SPSS statistical program (Chicago, Illinois, USA). P value as ≤ 0.05 was considered to indicate a statistically significant difference. Presence of significance in the four groups was once detected by LSD test. Withdrawal thresholds of Hot Plate and Von Frey were analyzed by using a Dixon non-parametric test. Remaining p value levels of significances in the data were analyzed by using Mann-Whitney U test.

### Compliance with Ethical Standards

The study was approved by the Local Experimental Animal Ethical Committee of Suleyman Demirel University (SDU) (Protocol number: HADYEK-21438139-320).
